# Study on the Magnetocaloric Effect of Room Temperature Magnetic Refrigerant Material La_0.5_Pr_0.5_(Fe_1−*x*_Co*_x_*)_11.4_Si_1.6_ and the Effect Arising from Co Doping on Its Curie Temperature

**DOI:** 10.3390/ma15041589

**Published:** 2022-02-20

**Authors:** Tao Shang, Lin Zheng, Jianjun Zhao, Guodong Li, Ruixia Wu

**Affiliations:** 1 Department of Physics Science and Technology, Baotou Teacher’s College, Baotou 014030, China; 66330@bttc.edu.cn (T.S.); zhenglin198172@126.com (L.Z.); 70183@bttc.edu.cn (R.W.); 2 Department of Physical Science and Technology, Inner Mongolia University, Hohhot 010021, China; lgd_pyre@163.com

**Keywords:** magnetocaloric effect, magnetic entropy change, magnetic refrigeration, Co doping, Curie temperature

## Abstract

The arc-melting method was adopted to prepare the compound La_0.5_Pr_0.5_(Fe_1−*x*_Co*_x_*)_11.4_Si_1.6_ (*x* = 0, 0.02, 0.04, 0.06, 0.08), and the magnetocaloric effect of the compound was investigated. As indicated by the powder X-ray diffraction (XRD) results, after receiving 7-day high temperature annealing at 1373 K, all the compounds formed a single-phase cubic NaZn_13_ crystal structure. As indicated by the magnetic measurement, the most significant magnetic entropy change |∆*S_M_*(*T*)| of the sample decreased from 28.92 J/kg·K to 4.22 J/kg·K with the increase of the Co content under the 0–1.5 T magnetic field, while the Curie temperature *T*_C_ increased from 185 K to the room temperature 296 K, which indicated that this series of alloys are the room temperature magnetic refrigerant material with practical value. By using the ferromagnetic Curie temperature theory and analyzing the effect of Co doping on the exchange integral of these alloys, the mechanism that the Curie temperature of La_0.5_Pr_0.5_(Fe_1−*x*_Co*_x_*)_11.4_Si_1.6_ and La_0.8_Ce_0.2_(Fe_1−*x*_Co*_x_*)_11.4_Si_1.6_ increased with the increase in the Co content was reasonably explained. Accordingly, this paper can provide a theoretical reference for subsequent studies.

## 1. Introduction

Room temperature magnetic refrigeration technology has aroused wide attention from numerous researchers for its high efficiency, energy saving and environmental protection. It is also a key issue to find an ideal ambient temperature refrigerant material for magnetic refrigeration research [1,2,3,4]. Compound LaFe_13−*x*_Si*_x_* has served as a potential room temperature magnetic refrigeration material because of its high magnetocaloric effect, low cost and environment-friendly property [5,6,7,8]. LaFe_13−*x*_Si*_x_* with a low Si content (*x* ≤ 1.6) has become the focus of this kind of compounds because of its large magnetic volume effect and the characteristic of itinerant electron magnetic transition above the Curie temperature *T*_C_ [9,10,11,12]. However, the Curie temperature of the LaFe_13−*x*_Si*_x_* compound with a low Si content is significantly lower than room temperature. Moreover, in order to improve the magnetic entropy change of the compound, other rare earth elements can be used to partially replace the La element in the compound, but this will further reduce the Curie temperature. This is very unfavorable to the practical application of the compound [13,14,15]. To address the mentioned problem, our research group has selected low-silicon compound LaFe_11.4_Si_6_ as the research object and prepared La_0.8_Ce_0.2_(Fe_1−*x*_Co*_x_*)_11.4_Si_1.6_ (*x* = 0.02, 0.04, 0.06) by partially replacing La and Fe with rare earth element Ce and transition metal Co [16]. As indicated by the experiment, the compound had a shorter annealing time of 7 days and a more significant magnetic entropy change. Moreover, the Curie temperature *T*_C_ of the sample increased from 207 K to 277 K with the increase in the Co content from *x* = 0.02 to *x* = 0.06. Thus, it was confirmed that Co doping could effectively increase the Curie temperature of the compound. However, the effect of Co doping on the Curie temperature of the compound has not been studied, and the discussion on the influencing mechanism in existing studies has been primarily limited to qualitative explanation, without thorough theoretical analysis.

Accordingly, a new intermetallic compound La_0.5_Pr_0.5_(Fe_1−*x*_Co*_x_*)_11.4_Si_1.6_ (*x* = 0, 0.02, 0.04, 0.06, 0.08) was prepared by partially replacing La and Fe with rare earth element Pr and transition metal Co. It is expected to obtain a La-Fe-Si compound with a Curie temperature reaching room temperature and a large magnetic entropy change. The crystal structure, magnetocaloric effect and magnetic transformation characteristics of La_0.5_Pr_0.5_(Fe_1−*x*_Co*_x_*)_11.4_Si_1.6_ compounds were studied. Moreover, by using the ferromagnetic Curie temperature theory and analyzing the effect of Co doping on the exchange integral of these alloys, the mechanism that the Curie temperature of La_0.5_Pr_0.5_(Fe_1−*x*_Co*_x_*)_11.4_Si_1.6_ and La_0.8_Ce_0.2_(Fe_1−*x*_Co*_x_*)_11.4_Si_1.6_ increased with the increase in the Co content was reasonably explained.

## 2. Materials and Methods

### 2.1. Fabrication

The purity of high pure metals La, Pr, Fe, Co and Si (General Research Institute for Nonferrous Metals, Beijing, China) was 99.0%. First, the materials were prepared in accordance with the chemical ratio of alloy La_0.5_Pr_0.5_(Fe_1−*x*_Co *_x_*)_11.4_Si_1.6_ (*x* = 0, 0.02, 0.04, 0.06, 0.08). Second, the raw material was placed into a WKDHL-II non-consumable electric arc furnace (Institute of Physics CAS, Beijing, China). After vacuumizing, argon gas (Hohhot Zirui Gas Co., Ltd, Hohhot, China) with a purity of 99.99% was injected into the arc furnace as the protective gas. The alloy was melted by a high-voltage arc. To make the melting uniform, the alloy at the molten state was stirred electromagnetically. After cooling, the sample was overturned and melted again 4–5 times. Subsequently, after melting and cooling, the alloy was clamped into small pieces, wrapped with molybdenum sheets (General Research Institute for Nonferrous Metals, Beijing, China) and then sealed in a quartz tube with a 3 × 10^−3^ Pa-high vacuum. High-temperature annealing was performed at 1373 K for 7 days, and then the high-temperature samples were directly placed into the mixture of ice water for rapid cooling to ambient temperature for quenching. Lastly, the desired compound was obtained.

### 2.2. Characterization

The annealed samples were crushed into powder while being protected by alcohol (Tianjin Chemical Plant, Tianjin, China), and the crystal structure of the sample was studied using a Dmax/3A X-ray diffractometer (Rigaku, Tokyo, Japan). Besides, a small piece of sample was nipped from the annealed alloy sample and then polished repeatedly into a ball with a diameter of nearly 2 mm and a mass of 20–30 mg. Lastly, the ball was loaded into the WKVSM vibrating sample magnetometer (Institute of Physics CAS, Beijing, China), and the parameters were set for the magnetic measurement.

## 3. Results

**Figure 1:**
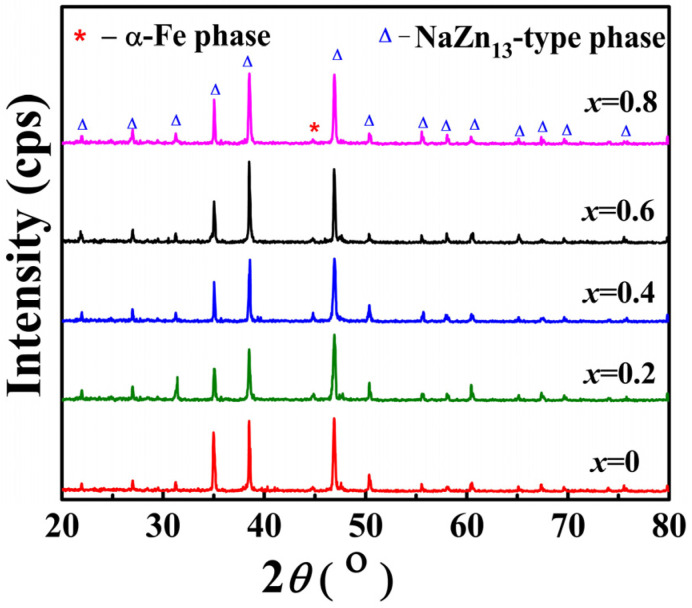
X-ray diffraction patterns of La_0.5_Pr_0.5_(Fe_1−*x*_Co*_x_*)_11.4_Si_1.6_.

Figure 1 shows X-ray diffraction (XRD) patterns of intermetallic compound powders after the 7-day annealing at ambient temperature. As indicated by the figure, except for a small amount of α-Fe impurity phase marked with *, all samples formed the main phase of cubic NaZn_13_ type. The NaZn_13_-type single phase was formed in *x* = 0 samples (samples without Co addition), thus indicating that the addition of Pr primarily accounted for the shortening of the annealing time of this series of compounds; it also shows that the samples maintain a short annealing phase-forming time after the addition of Co. Moreover, the diffraction peak of the sample after the addition of Co did not change significantly, and the superlattice diffraction peak of Co atoms was not identified in the diffraction diagram, which indicated that the doped Co atoms occupied the positions of 8b and 96i of Fe atoms randomly. In accordance with the XRD diffraction data, the main diffraction peaks of the compounds shift to a high angle with the increase of Co content. According to the calculation results, the lattice parameter tended to decrease from 1.1452 nm (*x* = 0) to 1.1408 nm. This can be attributed to the fact that the radius of the Co atom (1.26 nm) is smaller than that of the Fe atom (1.27 nm).

**Figure 2:**
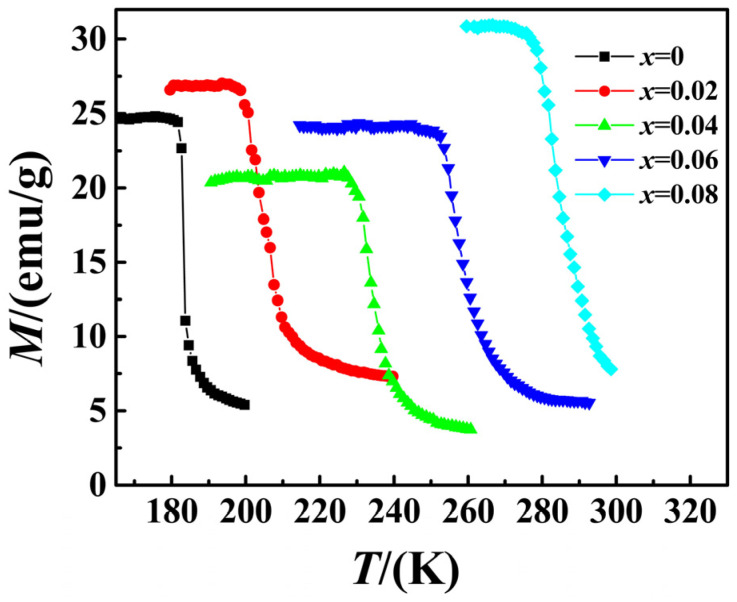
Thermomagnetic curves of annealed La_0.5_Pr_0.5_(Fe_1−*x*_Co*_x_*)_11.4_Si_1.6_.

Figure 2 illustrates the thermomagnetic curves of sample La_0.5_Pr_0.5_(Fe_1−*x*_Co*_x_*)_11.4_Si_1.6_ (*x* = 0, 0.02, 0.04, 0.06, 0.08) at a low magnetic field of 0.05 T, and the ferromagnetic Curie point *T*_C_ of the sample can be determined by the first derivative of the curve. As indicated by the figure, the Curie temperature *T*_C_ of the sample increased significantly with the increase in *x* value of the Co content. The Curie temperatures of the samples *x* = 0, 0.02, 0.04 and 0.06 were 185 K, 215 K, 241K and 267 K, respectively, and the Curie temperature of the sample *x* = 0.08 was 296 K, which reached room temperature. Accordingly, the sample had the potential of practical application. Furthermore, the magnetization intensity *M* of the respective sample changed significantly with temperature *T* near its *T*_C_, and it changed very significantly especially at *x* = 0, thus indicating that the sample had a significant magnetic entropy change.

**Figure 3:**
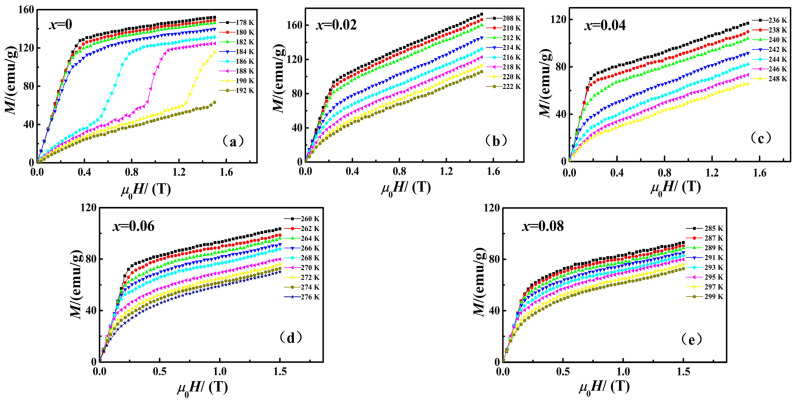
Magnetization curves (**a**–**e**) of La_0.5_Pr_0.5_(Fe_1−*x*_Co*_x_*)_11.4_Si_1.6_ (*x* = 0, 0.02, 0.04, 0.06, 0.08).

The isothermal magnetization curves of the samples with temperature intervals of 2 K near their respective Curie temperature *T*_C_ are presented in Figure 3. According to the figure, all samples had a ferromagnetic state when the temperature was lower than their *T_C_* and showed a paramagnetic state when the temperature was higher than the Curie temperature *T*_C_. As highlighted by existing studies, the La(Fe*_x_*Si_1−*x*_)_13_ alloy compound shows a magnetic field-induced paramagnetic state to ferromagnetic state transformation behavior above the Curie temperature *T*_C_, i.e., itinerant electron magnetic transformation (IEMT) [17,18,19]. As revealed by the figure, the *M*–*H* curves of the respective sample were still bent and showed a saturation trend above Curie temperature *T*_C_, which were correlated with IEMT [19]. Therefore, it can be inferred that the La_0.5_Pr_0.5_(Fe_1−*x*_Co*_x_*)_11.4_Si_1.6_ compound prepared in this paper might have a significant magnetic entropy change close to the Curie point.

**Figure 4:**
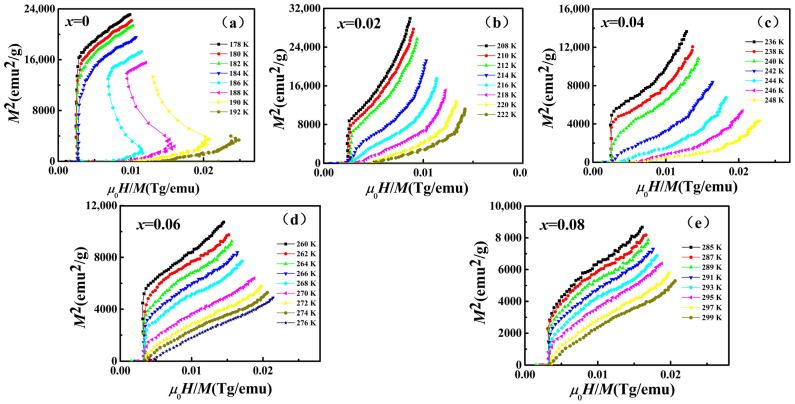
The Arrott curves (**a**–**e**) of La_0.5_Pr_0.5_(Fe_1−*x*_Co*_x_*)_11.4_Si_1.6_ (*x* = 0, 0.02, 0.04, 0.06, 0.08).

To further investigate the IEMT characteristics of La_0.5_Pr_0.5_(Fe_1−*x*_Co*_x_*)_11.4_Si_1.6_, the Arrott curves of the respective sample are presented in Figure 4. Using Ginzburg–Landau magnetic free energy expansion, Yamada et al. investigated the magnetic transformation behavior of magnetic materials [19,20]. They highlighted that when the magnetic field-induced itinerant electron magnetic transformation occurred, the fourth order coefficient of the material’s free energy expansion was negative; at this point, an inflection point would appear on the curve of its Arrott plot, or the slope of the curve would be negative [19,20]. According to the figure, the curve of the sample *x* = 0 exhibited an obvious negative slope at 186 K higher than its Curie temperature, while the negative slope characteristics of the curves with *x* = 0.02, 0.04, 0.06 and 0.08 above the Curie temperature gradually weaken with the increase of Co content. As indicated by the results, the magnetic field-induced itinerant electron magnetic transformation was identified when *x* = 0, and the IEMT characteristics of the samples gradually weaken with the increase in the Co content. This confirmed that replacing Fe atoms by Co atoms in the compound could inhibit the magnetic transformation. For the phase transformation properties, the IEMT is the characteristic of the first-order phase transition of the magnet. The IEMT characteristics of each sample gradually weaken with the increase of Co content, indicating that the magnetocaloric effect of the sample may be weakened.

Using the measured *μ*_0_*M*-*H* data curves and according to the Maxwell equation, the isothermal magnetic entropy change |∆*S_M_*(*T*)| of the sample was determined:

(1) $$\left|\Delta S_{M}\left(T,H\right)\right|={\int_{0}^{H}\left(\frac{\partial M}{\partial T}\right)}_{H}dH$$

**Figure 5:**
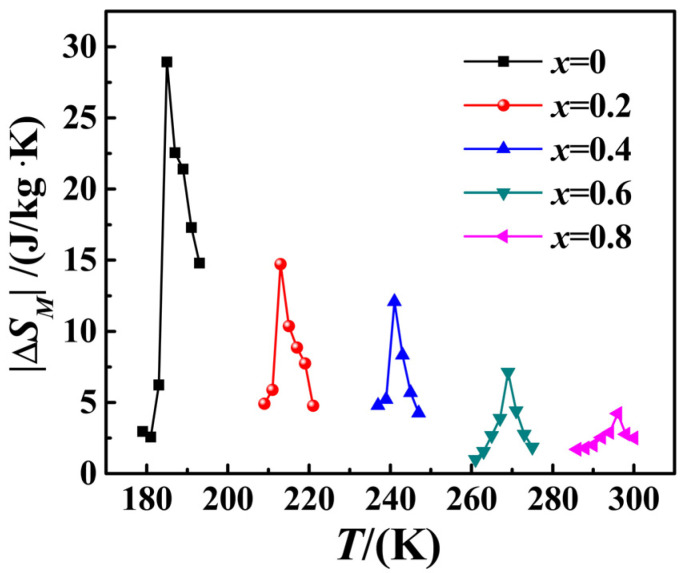
The isothermal magnetic entropy changes for La_0.5_Pr_0.5_(Fe_1−*x*_Co*_x_*)_11.4_Si_1.6_ with the magnetic field changes of 0–1.5 T.

Notably, Equation (1) is applicable to the second-order phase transition, whereas it should be cautious when applying to the first-order phase transition. However, Equation (1) has been proven to be suitable for the first-order phase transition of the La-Fe-Si alloy [21,22,23]. Figure 5 presents the curves of the absolute value of isothermal magnetic entropy change as a function of temperature for compound La_0.5_Pr_0.5_(Fe_1−*x*_Co*_x_*)_11.4_Si_1.6_ when the applied magnetic field was 0–1.5 T. According to the figure, when *x* = 0, 0.02 and 0.04, the peak values of the magnetic entropy change of the samples were obviously broadened to the high temperature region, and the peak value of the magnetic entropy change of the samples with different Co contents reached the maximum near the Curie temperature, and the |∆*S_M_*(*T*)| values of samples with *x* = 0, 0.02, 0.04, 0.06 and 0.08 were 28.92 J/kg·K, 14.72 J/kg·K, 12.10 J/kg·K, 7.12 J/kg·K and 4.22 J/kg·K, respectively. The reason for the large magnetic entropy peak of samples *x* = 0, 0.02 and 0.04 can be attributed to the first-order phase transition, with a large $\frac{\partial M}{\partial T}$ value at the phase transition point, accompanied by the magnetic volume effect and the IEMT close to the phase transformation temperature *T*_C_. With the increase in the Co content *x*, the magnetic entropy change of the samples when *x* = 0.06, 0.08 showed a *λ* shape with the temperature change, which could be considered a typical characteristic of the second-order phase transition. Thus, it was revealed that the phase transition property of the sample transited gradually from the first-order phase transition to the second-order phase transition, so it would also account for the decrease in the maximum magnetic entropy change of the sample.

In general, with the increase in the Co content, the Curie temperature *T*_C_ of the sample almost shows a linear increase. The Curie temperature *T*_C_ of the sample when *x* = 0.06 is 267 K, which is close to ambient temperature, and the sample kept a significant magnetic entropy change. The maximum magnetic entropy change |∆*S_M_*(*T*)| is 7.65 J/kg·K, which is far larger than the 3.1 J/kg·K of commercial-grade metal Gd [23]. Furthermore, the *T*_C_ of the sample when *x* = 0.08 was 296 K, which has reached room temperature, and the maximum|∆*S_M_*(*T*)| is 4.22 J/kg·K, which is better than that of commercial-grade Gd, proving that the alloy compounds have practical values. In addition, compared with similar materials, the maximum|∆*S_M_*(*T*)|of the compounds prepared was close to or lower than that of precious metal alloy Fe_49_Rh_51_ (approximately 8.0 J/kg·K of that prepared by slow cooling and about 13.0 J/kg·K of that prepared by rapid quenching). However, the Curie temperature of alloy Fe_49_Rh_51_ was too high (about 323 K), about 20 K higher than the ambient temperature, which revealed that the compounds prepared also had certain advantages [24].

## 4. Discussion

**Figure 6:**
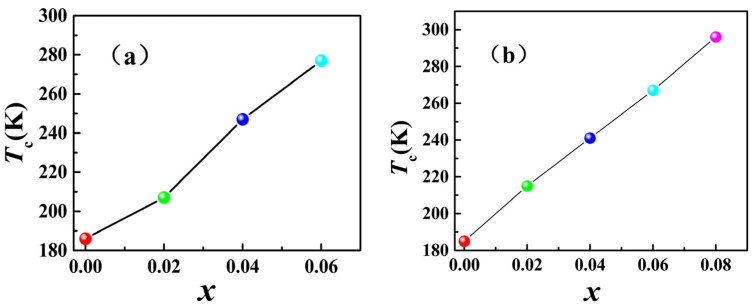
The Curve of Curie temperature *T_C_* of La_0.8_Ce_0.2_(Fe_1__−*x*_Co*_x_*)_11.4_Si_1.6_ (**a**) and La_0.5_Pr_0.5_(Fe_1__−*x*_Co*_x_*)_11.4_Si_1.6_ (**b**) with Co content *x*.

According to our existing studies [16] and the above experimental data, in both compounds La_0.8_Ce_0.2_(Fe_1−*x*_Co*_x_*)_11.4_Si_1.6_ and La_0.5_Pr_0.5_(Fe_1−*x*_Co*_x_*)_11.4_Si_1.6,_ the Curie temperature *T*_C_ increases significantly with the increase of the addition of Co. Figure 6 shows the changing curve of the Curie temperature *T*_C_ of two compounds with the Co content *x*. As indicated by the figure, the Curie temperature of the two compounds increases nearly linearly. In order to more obviously show the change law of the data, the linear equation is used to fit the experimental data. Equations (2) and (3) are the linear fitting equations of the Curie temperatures of two kinds of compounds, La_0.8_Ce_0.2_(Fe_1−*x*_Co*_x_*)_11.4_Si_1.6_ and La_0.5_Pr_0.5_(Fe_1−*x*_Co*_x_*)_11.4_Si_1.6_, with the Co content *x*, respectively.

*T*_C_ = 186 + 1500*x* (2)

*T*_C_ = 185 + 1366*x* (3)

Table 1 lists the experimental data and fitting data of Curie temperature *T*_C_. According to the table, the fitting data are well consistent with the experimental data, indicating that linear fitting Equations (2) and (3) can more effectively indicate the change trend of the Curie temperature with Co content.

The theory of ferromagnetic Curie temperature is presented below [25,26]. Weiss theory assumes that the atomic magnetic moment in a ferromagnet is not only dependent on the external magnetic field *H*, but also on the internal “molecular field” defined as $H_{m}=\lambda M$. The *λ* denotes the molecular field constant. On that basis, the Curie temperature of ferromagnet was deduced:

(4) $$T_{C}=\lambda \frac{NJ(J+1){g_{J}}^{2}{\mu_{B}}^{2}}{3k_{B}}$$

where *N* represents the number of atoms per unit volume; *J* denotes the total angular momentum quantum number. In 1928, Heisenberg proposed the exchange interaction between adjacent atoms and designed the molecular field constant *λ*. The expression is as follows:

(5) $$\lambda =\frac{2J_{e}Z}{N{g_{S}}^{2}{\mu_{B}}^{2}}$$

where *Z* denotes the coordination number; $J_{e}$ represents the exchange integral, indicating the exchange action. Substituting Equation (5) into Equation (4), since the atomic magnetic moment of ferromagnet was largely provided by spin, only spin was included (*J* = *S*). The Curie temperature can be written as

(6) $$T_{C}=\frac{2Z}{3}[S(S+1)]J_{e}$$

where *Z* denotes the coordination number; $J_{e}$ represents the exchange integral, indicating the exchange action. The exchange integral $J_{e}$ and *R*/*R*_d_ conform to the Bette Slater relationship curve, where *R* denotes the atomic radius and *R*_d_ represents the orbital radius of the magnetic electron. The Equation (6) is successful in the qualitative explanation of the Curie temperature magnetism of Fe, Co, Ni and other metals and their alloys. For instance, for a binary alloy, its exchange action could be expressed by the superposition of the exchange integral $J_{AB}$, $J_{AA}$ and $J_{BB}$ between different neighboring pairs of atoms. Thus, a relation similar to Equation (6) can be determined [25]:

(7) $$T_{C}=\frac{Z}{2k}({n_{A}}^{2}J_{AA}+{n_{B}}^{2}J_{BB}+2n_{B}n_{A}J_{AB})$$

where $J_{AB}$, $J_{AA}$ and $J_{BB}$ denote the exchange integral between pairs of neighboring atoms, and $n_{i}$ represents the percentage of the respective element in the alloy. In the LaFe_13−*x*_Si*_x_* compound, the number of electrons in the 4f orbital of La atom is zero, and its magnetic moment can be ignored. Accordingly, the Curie temperature of the compound was determined by the following factors: (1) ferromagnetic exchange interaction between Fe–Fe; (2) saturation magnetic moment of each Fe atom and (3) coordination number of Fe–Fe. As reported by existing studies [27,28,29], when the Si content was 1.2 ≤ *x* ≤ 2.6, the exchange interaction between Fe and Fe played a major role in determining the magnetic properties. The Si content of the two compounds in this paper is *x* = 1.6, between 1.2 ≤ *x* ≤ 2.6, so the exchange interaction between Fe–Fe played a major role in determining the magnetic properties. Moreover, since the Si content remained constant in the two compounds, the effect of Si on the magnetic change of the compound can be ignored.

First, the Curie temperature of compound LaFe_11.4_Si_1.6_ was studied after La was substituted with Ce or Pr. For compound LaFe_11.4_Si_1.6_, the magnetic moment of La could be ignored, so only the interaction between iron atoms was considered. According to Equation (7), the qualitative expression of the Curie temperature of the LaFe_11.4_Si_1.6_ compound can be written as

(8) $$T_{C}=\frac{Z}{2k}({n_{Fe}}^{2}J_{Fe-Fe})$$

where $J_{Fe-Fe}$ denotes the exchange integral between iron atoms, and $n_{Fe}$ represents the percentage of iron atoms in the compound. Since the atomic radius of Ce and Pr was both smaller than that of La, replacing La with Ce or Pr in LaFe_11.4_Si_1.6_ would reduce the cell volume and reduce the lattice constant of the compound. Moreover, according to the Bette Slater relationship, the exchange integral $J_{Fe-Fe}$ between iron atoms of iron-based compounds would decrease with the decrease of lattice constant. If ${J_{Fe-Fe}}^{\prime }$ represents the exchange integral between iron atoms in the compound after adding Ce or Pr, there is

(9) $${J_{Fe-Fe}}^{\prime }<J_{Fe-Fe}$$

Besides, since both Ce and Pr have atomic magnetic moments, the addition of Ce and Pr elements would introduce an interaction between the transition metal (TM) and rare earth (R) as well as an interaction between rare earth elements in the compound. The exchange integrals are expressed by $J_{R-T}$ and $J_{R-R}$ (R = Ce, Pr), respectively. Thus, there were mainly three commutative integrals, including $J_{T-T}$ (here is $J_{Fe-Fe}$), $J_{T-R}$ (here is $J_{Fe-R}$) and $J_{R-R}$ in the substituted compound. Moreover, since the substitution of Ce or Pr for La did not change the percentage of iron atoms, i.e., $n_{Fe}$ was not changed, the qualitative expression of the Curie temperature of the compound after substitution ${T_{C}}^{\prime }$ can be written as

(10) $${T_{C}}^{\prime }=\frac{Z}{2k}({n_{Fe}}^{2}{J_{Fe-Fe}}^{\prime }+{n_{R}}^{2}J_{R-R}+2n_{Fe}n_{R}J_{Fe-R})$$

where $n_{R}$ denotes the percentage of rare earth elements Ce or Pr. Numerous experiments [26] have shown that, in rare earth transition metal alloys, the value of $J_{T-T}$ (positive) is the largest followed by $J_{T-R}$ (negative) and $J_{R-R}$. The value of $J_{R-R}$ is very small, and its effect is shown only at low temperatures or when $J_{T-T}$ and $J_{T-R}$ are small, which can be ignored here. So, Equation (10) can be rewritten as

(11) $${T_{C}}^{\prime }=\frac{Z}{2k}({n_{Fe}}^{2}{J_{Fe-Fe}}^{\prime }-2n_{Fe}n_{R}\left|J_{Fe-R}\right|)$$

By comparing Equations (8) and (11) and considering Equation (9), the following equation can be yielded:

(12) $${T_{C}}^{\prime }<T_{C}$$

Therefore, substituting La with Ce or Pr would lead to a decrease in the Curie temperature of the compound, which is consistent with the experimental fact.

Subsequently, how the Curie temperature changed after Fe was substituted by a small amount of Co was analyzed. On the one hand, the effect arising from Co substitution on the original Fe–Fe interaction and Fe–R interaction was considered. As the radius of Co atoms is slightly smaller than that of Fe atoms, the substitution of Fe by Co would cause the cell volume to shrink slightly and the lattice constant to decrease slightly. This would result in a slight decrease in ${J_{Fe-Fe}}^{\prime }$. However, arising from the small amount of Co substitution, the reduction of the lattice constant of the compounds could be basically ignored, which could be confirmed in the previous X-ray diffraction diagram. Thus, the reduction of ${J_{Fe-Fe}}^{\prime }$ could be approximately ignored. In addition, the substitution of Co would also change $J_{Fe-R}$, and this change could also be ignored. If ${J_{Fe-Fe}}^{\prime \prime }$ and ${J_{Fe-R}}^{\prime }$ denoted the exchange integral between Fe–Fe atoms and Fe–R atoms in the compound after Co substitution, respectively, then it yields

(13) $${J_{Fe-Fe}}^{\prime \prime }\approx {J_{Fe-Fe}}^{\prime },{J_{Fe-R}}^{\prime }\approx J_{Fe-R}$$

On the other hand, the substitution of Co would introduce an Fe–Co interaction, Co–Co interaction and Co–R interaction into the compound. The exchange integrals were denoted $J_{Fe-Co}$, $J_{Co-Co}$ and $J_{Co-R}$, respectively. Then, ${n_{Fe}}^{\prime }$ and $n_{Co}$ were used to represent the percentage of Fe atoms and Co atoms in the compound after Co substitution. Moreover, it was noteworthy that Co entered the lattice as the replacement atoms of Fe, so $n_{R}$ did not change before and after Co substitution. The Curie temperature ${T_{C}}^{\prime \prime }$ of the compound after replacing Fe with Co can have the qualitative expression below:

(14) $$\begin{array}{ll} {T_{C}}^{\prime \prime }= & \frac{Z}{2k}({n_{Fe}}^{\prime 2}{J_{Fe-Fe}}^{\prime \prime }+{n_{Co}}^{2}J_{Co-Co}+2{n_{Fe}}^{\prime }n_{Co}J_{Fe-Co}+2n_{Co}n_{R}J_{Co-R}\\ & -2{n_{Fe}}^{\prime }n_{R}\left|{J_{Fe-R}}^{\prime }\right|) \end{array}$$

The above formula was further analyzed. First, the Co content was small, and the content of rare earth element R that replaced La was also small. In addition, the value of $J_{T-R}$ itself is relatively small, so the Co-R interaction could be ignored, i.e., $J_{Co-R}$ could be ignored. Thus, the above formula can be written as

(15) $${T_{C}}^{\prime \prime }=\frac{Z}{2k}({n_{Fe}}^{\prime 2}{J_{Fe-Fe}}^{\prime \prime }+{n_{Co}}^{2}J_{Co-Co}+2{n_{Fe}}^{\prime }n_{Co}J_{Fe-Co}-2{n_{Fe}}^{\prime }n_{R}\left|{J_{Fe-R}}^{\prime }\right|)$$

Second, it is known that the value of the exchange integral J of transition elements increases with the increase of the atomic number *Z* [26]. So, it can be concluded that the Co–Co interaction should be higher than the Fe–Fe interaction, which is expressed below:

(16) $$J_{Co-Co}>{J_{Fe-Fe}}^{\prime \prime }$$

Moreover, since the Co atom has one more 3d electron than the Fe atom, and the *R*/*R*_d_ value of Co was 3.64, significantly higher than that of Fe 3.26 [26], it was inferred that $J_{Fe-Co}$ should also be higher than ${J_{Fe-Fe}}^{\prime \prime }$:

(17) $$J_{Fe-Co}>{J_{Fe-Fe}}^{\prime \prime }\approx {J_{Fe-Fe}}^{\prime }$$

It is noteworthy that Co, Ce or Pr entered the lattice as the substituted atoms, and Co was substituted for Fe, so it yields

(18) $$n_{Fe}={n_{Fe}}^{\prime }+n_{Co}=\mathrm{Constant}$$

Accordingly,

(19) $${n_{Fe}}^{2}={n_{Fe}}^{\prime 2}+{n_{Co}}^{2}+2{n_{Fe}}^{\prime }n_{Co}=\mathrm{Constant}$$

Considering Equations (13), (16), (17) and (19), the following equation can be yielded:

(20) $${n_{Fe}}^{\prime 2}{J_{Fe-Fe}}^{\prime \prime }+{n_{Co}}^{2}J_{Co-Co}+2{n_{Fe}}^{\prime }n_{Co}J_{Fe-Co}>{n_{Fe}}^{2}{J_{Fe-Fe}}^{\prime }$$

Since $n_{Fe}={n_{Fe}}^{\prime }+n_{Co}$, i.e., $n_{Fe}>{n_{Fe}}^{\prime }$ and $n_{R}$ has not changed after Co substitution, considering Equation (13), we can obtain $2{n_{Fe}}^{\prime }n_{R}\left|{J_{Fe-R}}^{\prime }\right|<2n_{Fe}n_{R}\left|J_{Fe-R}\right|$; that is,

(21) $$-2{n_{Fe}}^{\prime }n_{R}\left|{J_{Fe-R}}^{\prime }\right|>-2n_{Fe}n_{R}\left|J_{Fe-R}\right|$$

Add Equations (20) and (21) and multiply by $\frac{Z}{2k}$ on both sides of the inequality sign to obtain

(22) $${T_{C}}^{\prime \prime }>{T_{C}}^{\prime }$$

Thus, the mechanism that the Curie temperature of the compound increased after Fe was replaced with a small amount of Co was explained.

Lastly, how the Curie temperature ${T_{C}}^{\prime \prime }$ would change when the Co substitution amount further increased was analyzed. When the Co substitution quantity increased, the percentage of Co atoms $n_{Co}$ would increase. According to Equation (18), when $n_{Co}$ increased, ${n_{Fe}}^{\prime }$ would be reduced. Thus, among the first three terms ${n_{Fe}}^{\prime 2}{J_{Fe-Fe}}^{\prime \prime }+{n_{Co}}^{2}J_{Co-Co}+2{n_{Fe}}^{\prime }n_{Co}J_{Fe-Co}$ of Equation (15), the proportion of the contribution of the second and third terms to the Curie temperature increased, while the proportion of the first item would decrease. Considering Equations (16), (17) and (19), it was concluded that ${n_{Fe}}^{\prime 2}{J_{Fe-Fe}}^{\prime \prime }+{n_{Co}}^{2}J_{Co-Co}+2{n_{Fe}}^{\prime }n_{Co}J_{Fe-Co}$ would increase. Next, considering that the fourth term $-2{n_{Fe}}^{\prime }n_{R}\left|{J_{Fe-R}}^{\prime }\right|$ in Equation (15), and as $n_{Co}$ increases, ${n_{Fe}}^{\prime }$ decreases while $n_{R}$ remains unchanged, $-2{n_{Fe}}^{\prime }n_{R}\left|{J_{Fe-R}}^{\prime }\right|$ would increase. In general, it can be obtained that all four terms in Equation (15) increase with the increase of $n_{Co}$. In other words, the Curie temperature ${T_{C}}^{\prime \prime }$ of the compound increased rapidly with the increase in the Co substitution amount, which is consistent with the experimental fact.

Moreover, according to the fitting of Equations (2) and (3), the slope of Equation (2) is 1500, which larger than that of Equation (3), 1366. It indicated that Co could more significantly improve the Curie temperature of compound La_0.8_Ce_0.2_(Fe_1__−_*_x_*Co*_x_*)_11.4_Si_1.6_ than it could of compound La_0.5_Pr_0.5_(Fe_1−*x*_Co*_x_*)_11.4_Si_1.6_. This could be attributed to the fact that $J_{Co-R}$ has been previously overlooked by us. As explained above, $J_{T-R}$ has a small and negative value in the compound. Here, if $J_{T-R}$, including the Curie temperature can be written according to Equation (14) as

(23) $${T_{C}}^{\prime \prime \prime }=\frac{Z}{2k}({n_{Fe}}^{\prime 2}{J_{Fe-Fe}}^{\prime \prime }+{n_{Co}}^{2}J_{Co-Co}+2{n_{Fe}}^{\prime }n_{Co}J_{Fe-Co}-2{n_{Fe}}^{\prime }n_{R}\left|{J_{Fe-R}}^{\prime }\right|-2n_{Co}n_{R}\left|J_{Co-R}\right|)$$

After substituting Equation (15) into the above equation, the following equation can be yielded:

(24) $${T_{C}}^{\prime \prime \prime }={T_{C}}^{\prime \prime }-\frac{Z}{2k}n_{Co}n_{R}\left|J_{Co-R}\right|$$

${T_{C}}^{\prime \prime }$ is the major contribution of Curie temperature ${T_{C}}^{\prime \prime \prime }$. As revealed by the above analysis, ${T_{C}}^{\prime \prime }$ increases rapidly with the increase in the Co substitution amount. While the second term $-\frac{Z}{2k}n_{Co}n_{R}\left|J_{Co-R}\right|$ in Equation (15) is negative, its absolute value increases with the increase of the percentage of Co atoms $n_{Co}$. Therefore, the effect of this term can slow down the increase speed of ${T_{C}}^{\prime \prime }$ with the increase in the Co substitution amount. When same amount of Co atoms were added to the two types of compounds, the $n_{Co}$ turned out to be $n_{Co}+\Delta n_{Co}$, $J_{Co-Pr}$ and $J_{Co-Ce}$ became $J_{Co-Pr}+\Delta J_{Co-Pr}$ and $J_{Co-Ce}+\Delta J_{Co-Ce}$, while $n_{Pr}$ and $n_{Ce}$ remained unchanged. Accordingly, for the two types of compounds, the term $-\frac{Z}{2k}n_{Co}n_{R}\left|J_{Co-R}\right|$ can be written as

(25) $$-\frac{Z}{2k}(n_{Co}+\Delta n_{Co})n_{Pr}\left|\left(J_{Co-Pr}+\Delta J_{Co-Pr}\right)\right|,$$

(26) $$-\frac{Z}{2k}(n_{Co}+\Delta n_{Co})n_{Ce}\left|\left(J_{Co-Ce}+\Delta J_{Co-Ce}\right)\right|$$

After the above two equations are simplified, the following equations can be yielded:

(27) $$-\frac{Z}{2k}n_{Pr}(n_{Co}\left|J_{Co-Pr}\right|+n_{Co}\left|\Delta J_{Co-Pr}\right|+\Delta n_{Co}\left|J_{Co-Pr}\right|+\Delta n_{Co}\left|\Delta J_{Co-Pr}\right|)$$

(28) $$-\frac{Z}{2k}n_{Ce}(n_{Co}\left|J_{Co-Ce}\right|+n_{Co}\left|\Delta J_{Co-Ce}\right|+\Delta n_{Co}\left|J_{Co-Ce}\right|+\Delta n_{Co}\left|\Delta J_{Co-Ce}\right|)$$

assuming

(29) $$\Delta nJ(Co-Pr)=n_{Co}\left|\Delta J_{Co-Pr}\right|+\Delta n_{Co}\left|J_{Co-Pr}\right|+\Delta n_{Co}\left|\Delta J_{Co-Pr}\right|$$

(30) $$\Delta nJ(Co-Ce)=n_{Co}\left|\Delta J_{Co-Ce}\right|+\Delta n_{Co}\left|J_{Co-Ce}\right|+\Delta n_{Co}\left|\Delta J_{Co-Ce}\right|$$

Subsequently, Equations (27) and (28) can be written as

(31) $$-\frac{Z}{2k}n_{Pr}n_{Co}\left|J_{Co-Pr}\right|-\frac{Z}{2k}n_{Pr}\Delta nJ(Co-Pr)$$

(32) $$-\frac{Z}{2k}n_{Ce}n_{Co}\left|J_{Co-Ce}\right|-\frac{Z}{2k}n_{Ce}\Delta nJ(Co-Ce)$$

where $\frac{Z}{2k}n_{Pr}\Delta nJ(Co-Pr)$ and $\frac{Z}{2k}n_{Ce}\Delta nJ(Co-Ce)$ represent the reduction of the second term in Equation (24) after adding the same amount of Co atoms to the two compounds. In the two kinds of compounds, the content of rare earth element Pr (0.5) is relatively larger than that of Ce (0.2), so $n_{Pr}$ > $n_{Ce}$. In addition, the Pr atom has one more 4f electron than does the Ce atom, so $\left|J_{Co-Pr}\right|$ > $\left|J_{Co-Ce}\right|$ and $\left|\Delta J_{Co-Pr}\right|$ > $\left|\Delta J_{Co-Ce}\right|$. Therefore, by comparing Equations (29) and (30), the conclusion that $n_{Pr}\Delta nJ(Co-Pr)$ > $n_{Ce}\Delta nJ(Co-Ce)$ can be obtained so that

(33) $$\frac{Z}{2k}n_{Pr}\Delta nJ(Co-Pr)>\frac{Z}{2k}n_{Ce}\Delta nJ(Co-Ce)$$

This indicates that adding the same amount of Co atoms to La_0.5_Pr_0.5_(Fe_1−*x*_Co*_x_*)_11.4_Si_1.6_ would lead to a greater reduction in the second term of the compound in Equation (24) than that of La_0.5_Ce_0.2_(Fe_1−*x*_Co*_x_*)_11.4_Si_1.6_. Thus, it has a stronger effect on slowing down the rising speed of ${T_{C}}^{\prime \prime }$ increases in La_0.5_Pr_0.5_(Fe_1−*x*_Co*_x_*)_11.4_Si_1.6_. In brief, when the Co content added to the two compounds was the same, Co would more significantly improve the Curie temperature of La_0.8_Ce_0.2_(Fe_1−*x*_Co*_x_*)_11.4_Si_1.6_ than that of La_0.5_Pr_0.5_(Fe_1−*x*_Co*_x_*)_11.4_Si_1.6_. In other words, the slope of Equation (2) is higher than that of Equation (3).

## 5. Conclusions

In this paper, compound La_0.5_Pr_0.5_(Fe_1−*x*_Co*_x_*)_11.4_Si_1.6_ (*x* = 0, 0.02, 0.04, 0.06, 0.08) was prepared using the arc-melting method, and the magnetic properties of the compound were investigated. As revealed by the powder X-ray diffraction results, compound La_0.5_Pr_0.5_(Fe_1−*x*_Co*_x_*)_11.4_Si_1.6_ formed a single-phase cubic NaZn_13_ crystal structure after being annealed at 1373 K in a vacuum for 7 days. With the increase in the Co content from *x* = 0 to *x* = 0.08, the Curie temperature *T*_C_ of the sample rose from 185 K to 296 K, room temperature. Under 0–1.5 T magnetic field, the maximal magnetic entropy change |∆*S_M_*(*T*)| of the sample when *x* = 0, 0.02, 0.04, 0.06 and 0.08 was determined as 28.92 J/kg·K, 14.72 J/kg·K, 12.10 J/kg·K, 7.12 J/kg·K and 4.22 J/kg·K, respectively, which are all better than for the metal Gd, indicating that the alloy could be a room-temperature magnetic refrigeration material with practical values.

The effect arising from Co doping on the exchange integral in alloy was analyzed in accordance with the ferromagnetic Curie temperature theory. The influencing mechanism of substituting Fe with Co on the Curie temperature of the compounds La_0.8_Ce_0.2_(Fe_1−*x*_Co*_x_*)_11.4_Si_1.6_ (*x* = 0.02, 0.04, 0.06) and La_0.5_Pr_0.5_(Fe_1−*x*_Co*_x_*)_11.4_Si_1.6_ (*x* = 0, 0.02, 0.04, 0.06, 0.08) was explored. A conclusion was drawn that the change of lattice constant and interatomic interaction primarily impacted Curie temperature after Fe was substituted with Co. Thus, this paper provides a theoretical reference for subsequent studies.

## Figures and Tables

**Table 1:** The measured and the linear fitting Curie temperature of La_0.8_Ce_0.2_(Fe_1−*x*_Co*_x_*)_11.4_Si_1.6_ and La_0.5_Pr_0.5_(Fe_1−*x*_Co*_x_*)_11.4_Si_1.6_.

Compound	Collection Method	Co Content *x*				
		0	0.02	0.04	0.06	0.08
La_0.8_Ce_0.2_(Fe_1−*x*_Co*_x_*)_11.4_Si_1.6_	Measured *T*_C_/K	186	207	247	277	-
	Fitting *T*_C_/K	186	216	246	276	-
La_0.5_Pr_0.5_(Fe_1−*x*_Co*_x_*)_11.4_Si_1.6_	Measured *T*_C_/K	185	215	241	267	296
	Fitting *T*_C_/K	185	212	240	266	298

## Data Availability

The data presented in this study are available on request from the corresponding author.
